# Asymmetric Dimethylarginine *versus* Proton Pump Inhibitors Usage in Patients with Stable Coronary Artery Disease: A Cross-Sectional Study

**DOI:** 10.3390/ijms17040454

**Published:** 2016-04-15

**Authors:** Olga Kruszelnicka, Jolanta Świerszcz, Jacek Bednarek, Bernadeta Chyrchel, Andrzej Surdacki, Jadwiga Nessler

**Affiliations:** 1 Department of Coronary Artery Disease and Heart Failure, Jagiellonian University Medical College and John Paul II Hospital, 80 Prądnicka, 31-202 Cracow, Poland; jnessler@interia.pl; 2 Second Department of Cardiology, Jagiellonian University Medical College and University Hospital, 17 Kopernika, 31-501 Cracow, Poland; grasshoppers@interia.eu (J.Ś.); chyrchelb@gmail.com (B.C.); surdacki.andreas@gmx.net (A.S.); 3 Department of Electrocardiology, John Paul II Hospital, 80 Prądnicka, 31-202 Cracow, Poland; bednareks@op.pl

**Keywords:** asymmetric dimethylarginine, coronary artery disease, proton pump inhibitors

## Abstract

A recent experimental study suggested that proton pump inhibitors (PPI), widely used to prevent gastroduodenal complications of dual antiplatelet therapy, may increase the accumulation of the endogenous nitric oxide synthesis antagonist asymmetric dimethylarginine (ADMA), an adverse outcome predictor. Our aim was to assess the effect of PPI usage on circulating ADMA in coronary artery disease (CAD). Plasma ADMA levels were compared according to PPI use for ≥1 month prior to admission in 128 previously described non-diabetic men with stable CAD who were free of heart failure or other coexistent diseases. Patients on PPI tended to be older and with insignificantly lower estimated glomerular filtration rate (GFR). PPI use was not associated with any effect on plasma ADMA (0.51 ± 0.11 (SD) *vs.* 0.50 ± 0.10 µmol/L for those with PPI (*n* = 53) and without PPI (*n* = 75), respectively; *p* = 0.7). Additionally, plasma ADMA did not differ between PPI users and non-users stratified by a history of current smoking, CAD severity or extent. The adjustment for patients’ age and GFR did not substantially change the results. Thus, PPI usage does not appear to affect circulating ADMA in non-diabetic men with stable CAD. Whether novel mechanisms of adverse PPI effects on the vasculature can be translated into clinical conditions, requires further studies.

## 1. Introduction

Proton pump inhibitors (PPI)—widely used to prevent gastroduodenal complications of dual antiplatelet therapy—have recently been demonstrated to raise intracellular levels of asymmetric dimethylargininie (ADMA), an endogenous inhibitor of nitric oxide (NO) synthesis, which was accompanied by a lower NO formation, depressed endothelium-mediated vasorelaxation *in vitro* and increased circulating ADMA by about 20% in mice. These effects were ascribed to a PPI-dependent direct inhibition of the activity of the major ADMA-degrading enzyme type 1 dimethylarginine dimethylaminohydrolase (DDAH-1) [1].

Because ADMA is a recognized adverse outcome predictor in coronary artery disease (CAD) patients [2,3,4], the PPI‒ADMA interaction might contribute to an excessive cardiovascular risk in patients on PPI irrespective of the use of antiplatelet agents including clopidogrel, or a prior history of myocardial infarction [5,6,7,8,9,10,11]. Importantly, an elevated risk of myocardial infarction was associated with the usage of PPI but not H_2_-receptor antagonists also in the general population subjects, mainly without aspirin or clopidogrel, which may suggest an underlying mechanism not directly involving either platelet aggregation or changed drug absorption due to a rise in gastric pH [11,12]. Admittedly, potential negative clinical impacts of PPI on the risk of adverse cardiovascular events are still controversial [13] with conflicting results between randomized trials and observational studies [10,14]. Nevertheless, the proposed mechanistic concept [1] was not confirmed in a recent placebo-controlled, open-label, cross-over study where PPI administration for four weeks was not associated with significant effects on plasma ADMA or flow-dependent vasodilation in adults [15].

Therefore, our aim was to estimate the effect of PPI usage on circulating ADMA in stable CAD.

## 2. Results

Clinical and angiographic characteristics according to PPI use are shown in Table 1. Patients taking a PPI prior to admission (mainly omeprazole 20 mg o.i.d. or pantoprazole 20 mg o.i.d.) tended to be older and with lower estimated glomerular filtration rate (GFR).

The use of PPI was not associated with any effect on plasma ADMA (Table 2). In addition, there were no interactions between PPI use and the categorized potential confounders, *i.e.*, current smoking, angiographic CAD severity or extent in terms of plasma ADMA (*p* > 0.3), so that ADMA levels did not differ between PPI users and PPI non-users stratified by a history of current smoking, the presence of multivessel CAD or an over-median Sullivan score of angiographic CAD extent (Table 2). Adjustment for patients’ age and GFR by means of ANCOVA did not substantially change the results.

## 3. Discussion

Our salient finding was a similar plasma level of ADMA in PPI users and non-users. This observation appears inconsistent with the previously reported ability of PPI to augment ADMA accumulation *in vitro* and in an animal model through a direct inhibition of DDAH-1 [1], an enzyme influencing circulating ADMA [16,17,18,19]. On the other hand, in subjects with a history of vascular disease, Ghebremariam *et al.* [15] observed a more pronounced trend towards higher ADMA while on PPI compared to placebo in an interventional cross-over study, nevertheless the differences did not reach the statistical significance, which is in agreement with our cross-sectional retrospective analysis. To the best of our knowledge, our study is the second clinical report on ADMA levels in relation to PPI use.

### 3.1. Mechanistic Considerations

There are several potential explanations of these apparent discrepancies. First, all the patients were receiving angiotensin-converting enzyme inhibitors (ACEI), aspirin and statins, all of which had been previously shown to lower ADMA levels [20,21,22], thereby obscuring the putative influence of PPI on ADMA. On the other hand, ADMA concentrations in our patients were only slightly lower than ADMA levels measured by the same enzyme-linked immunosorbent assay (ELISA) in control groups of largely untreated subjects of similar age from European populations and without evidence of atherosclerotic vascular disease [23,24,25], which argues against the proposed explanation and strengthens our findings.

Second, even if the PPI-DDAH-1 interaction took place *in vivo*, its effects on plasma ADMA could be attenuated or nullified by an effective counter-regulatory mechanism. This hypothetical mechanism might involve any of the recognized determinants of circulating ADMA levels including DDAH-mediated ADMA degradation, urinary ADMA excretion, the activity of type I protein-arginine *N*-methyltransferases (PRMTs-I), proteolysis rate of proteins with dimethylated arginine residues, and interorgan ADMA transport [26]. Of note, Becker *et al.* [27] described depressed nicotinamide adenine dinucleotide phosphate (NADPH)-dependent superoxide release and augmented expression of the antioxidant defense enzyme type 1 heme oxygenase (HO-1) in human endothelial cells exposed for 8–24 h to lansoprazole at final concentrations as low as 30 µmol/L, *i.e.*, similar to PPI levels (20 µmol/L) that increased intracellular ADMA concentrations by about 30% via DDAH-1 inhibition as shown by Ghebremariam *et al.* [1]. The PPI-dependent HO-1 induction occurred at the level of transcription [27], in contrast to PPI direct effects on DDAH-1 activity [1], which can probably further potentiate the former effect in subjects on chronic PPI therapy. Accordingly, because oxidative stress stimulates PRMTs-I expression [28] and inhibits DDAH activity [29,30], the PPI-mediated decrease in endothelial superoxide formation [27] could possibly indirectly downregulate ADMA formation and enhance ADMA degradation, thus counteracting the ADMA-increasing effect of the direct DDAH-1 inhibition by PPI [1].

Third, findings from animal experiments cannot be simply extrapolated to clinical conditions because the presence of atherosclerotic cardiovascular disease and risk factors may interfere with ADMA-regulating pathways. Nevertheless, in our hands, there were no significant interactions between PPI use and angiographic CAD extent or severity in terms of plasma ADMA and the results did not substantially change upon exclusion of current smokers from the analysis.

### 3.2. Study Limitations

First, a retrospective study design constrains conclusions drawn from our data. Second, our findings would be strengthened if we also assessed characteristics previously linked to adverse cardiovascular effects of PPI, *i.e.*, magnesium or homocysteine (due to a putative PPI-induced vitamin B_12_ deficiency), and platelet response to aspirin (attributable to reduced aspirin absorption at a higher intragastric pH). Nevertheless, chronic PPI therapy is unlikely to induce clinically relevant changes in serum magnesium [31], vitamin B_12_ or homocysteine [32]. With regard to aspirin antiplatelet effect, contradictory results were reported in patients on a low-dose aspirin treated with concomitant PPI [33,34] and no involvement of ADMA in this interaction [33] has been demonstrated so far. Third, coexistent diseases could affect our results, although we applied a wide range of exclusion criteria to limit the heterogeneity of the study population. Finally, PPI pharmacokinetics is profoundly modulated by genetic loss-of-function polymorphisms of cytochrome P450 (CYP) 2C19 isoform. Compared to so-called extensive metabolizers with both wild-type CYP2C19 alleles, poor mobilizers (those with both mutated CYP2C19 alleles) exhibit elevated circulating PPI levels, e.g., after oral omeprazole its peak plasma level was about 5-fold higher and the area under the concentration-time curve approximately 9-fold higher [35]. Admittedly, we did not perform either genetic or epigenetic testing. However, our aim was to compare ADMA in relation to PPI use in real-world clinical practice irrespective of genotype status. Additionally, the frequency of CYP2C19 poor metabolizers in Caucasian populations averages only about 2%–3% [36,37].

## 4. Materials and Methods

### 4.1. Patients

We performed an additional analysis of the dataset including ADMA levels and clinical and angiographic characteristics of stable CAD men who had previously been described [38]. The study subjects were free of heart failure or diabetes and exhibited the presence of ≥1 significant epicardial coronary stenosis on elective coronary angiography in our tertiary-care center [38]. All the patients were receiving a low-dose aspirin, ACEI and statin for at least 3 months prior to the hospitalization. As described previously [38], a wide set of exclusion criteria had been applied, including significant valvular heart disease, infections within previous 2 months, relevant coexistent diseases (e.g., severe renal insufficiency) and chronic non-cardiovascular medication with non-selective non-steroidal anti-inflammatory drugs or coxibs. Out of 151 CAD patients 23 were excluded from the current analysis due to missing data with regard to PPI use for ≥1 month before the index hospitalization.

In line with the Declaration of Helsinki, the study protocol was approved by the Bioethics Committee of the Jagiellonian University (Approval numbers: KBET/63/B/2006 dated 27 April 2006 and KBET/364/B/2012 dated 20 December 2012) and informed consent was obtained from the patients, as mentioned previously [38].

### 4.2. Procedure

A sample of peripheral venous blood was collected into ethylenediaminetetraacetic acid tubes in the fasting state in the morning prior to coronary angiography and plasma was kept frozen at −70 °C for subsequent biochemical analyses.

ADMA levels were measured by a commercially available ELISA (DLD Diagnostika GmbH., Hamburg, Germany)—as reported in detail [38]—and compared between 2 subgroups of the study subjects divided on the basis of a history of PPI use for ≥1 month before blood sampling on admission for ADMA assay. In addition, we compared ADMA levels in PPI users and non-users according to a history of current smoking, angiographic CAD severity (multivessel *vs.* one-vessel CAD) [39] and CAD extent quantified by means of the Sullivan score representing a percentage of the vessels with vascular wall irregularities on coronary angiography [40].

### 4.3. Statistical Analysis

Data have been presented as means ± SD (standard deviation) or medians (interquartile range) for continuous characteristics with normal and non-normal distribution, respectively. The concordance with a Gaussian distribution was checked by the Lilliefors’ test. The patients were compared according to PPI use by 2-tailed Student’s *t*-test or Mann-Whitney *U* test, and chi-squared test for continuous and categorical characteristics, respectively. According to a *post hoc* power calculation for the study group as a whole, the study design allowed to detect a difference in plasma ADMA between PPI users (*n* = 53) and non-users (*n* = 75) of 0.05 µmol/L (0.5 SD) with a power of 80% at a type I error rate of 0.05.

In order to test whether an effect of PPI use on circulating ADMA levels was modified by selected categorized covariates, a two-way analysis of variance (ANOVA) was performed to assess these potential interactions with plasma ADMA as a dependent variable and 2 independent factors: PPI use on the one hand and—on the other hand—a history of self-reported current smoking or angiographic CAD severity (multivessel *vs.* one-vessel CAD) or dichotomized CAD extent (an over-median (>29) *vs.* below-median (≤29) Sullivan score) as a coexistent factor; then an interaction between these factors was estimated. In addition, analysis of covariance (ANCOVA) was used to adjust for continuous clinical characteristics, for which the *p*-value in a univariate comparison between patients with and without PPI did not exceed 0.15. A *p*-value below 0.05 was inferred significant.

## 5. Conclusions

Thus, our preliminary cross-sectional findings suggest that PPI use does not appear to considerably affect circulating ADMA in non-diabetic men with stable CAD. Whether novel mechanisms of adverse PPI effects on the vasculature can be translated into clinical conditions, requires validation in large well-designed studies.

## Figures and Tables

**Table 1:** Characteristics of CAD patients according to PPI use prior to admission on a background of concomitant low-dose aspirin, ACEI and statin.

Characteristic	Patients on PPI (*n* = 53)	Patients without PPI (*n* = 75)	*p*-Value
Age (years)	59 ± 11	56 ± 10	0.12
Body-mass index (kg/m^2^)	27.7 ± 3.6	27.4 ± 3.5	0.6
History of current smoking, *n* (%)	16 (30%)	20 (27%)	0.8
Multivessel CAD, *n* (%)	41 (77%)	54 (72%)	0.6
CAD extent score	31 (21–44)	28 (19–40)	0.5
Left ventricular ejection fraction (%)	70 ± 7	68 ± 6	0.2
Hypertension, *n* (%)	43 (80%)	56 (75%)	0.4
Mean blood pressure (mm Hg)	96 ± 11	95 ± 10	0.7
Estimated GFR (mL/min per 1.73 m^2^)	69 ± 9	72 ± 11	0.09
LDL cholesterol (mmol/L)	2.8 ± 0.7	2.8 ± 0.6	0.8
HDL cholesterol (mmol/L)	0.9 ± 0.3	1.0 ± 0.3	0.2
Triglycerides (mmol/L)	1.4 ± 0.6	1.5 ± 0.7	0.3
Glucose (mmol/L)	5.8 ± 0.9	5.7 ± 0.8	0.5
High-sensitivity C-reactive protein (mg/L)	1.9 (1.1–4.0)	1.8 (1.0–3.8)	0.8

Data are shown as mean ± SD, median (interquartile range) or *n* (%); *p*-values by 2-tailed Student’s *t*-test or Mann-Whitney *U* test, and chi-squared test for proportions. CAD: coronary artery disease; ADMA: asymmetric dimethylarginine; ACEI: angiotensin-converting enzyme inhibitors; GFR: glomerular filtration rate calculated according to the Modification of Diet in Renal Disease study formula; HDL: high-density lipoproteins; LDL: low-density lipoproteins; PPI: proton pump inhibitors.

**Table 2:** Plasma ADMA levels according to PPI use prior to admission.

	ADMA before Admission (µmol/L)		*p*-Value
	PPI Users (*n* = 53)	PPI Non-Users (*n* = 75)	
All CAD subjects, *n* = 128	0.51 ± 0.11	0.50 ± 0.10	0.7
History of current smoking			
Yes, *n* = 36	0.51 ± 0.11	0.50 ± 0.10	0.4
No, *n* = 92	0.51 ± 0.10	0.51 ± 0.11	0.8
Severity of angiographic CAD			
One-vessel disease, *n* = 33	0.48 ± 0.10	0.49 ± 0.10	0.7
Multivessel disease, *n* = 95	0.52 ± 0.11	0.51 ± 0.11	0.9
Extent of angiographic CAD			
Sullivan extent score ≤ 29, *n* = 65	0.48 ± 0.09	0.49 ± 0.10	0.6
Sullivan extent score > 29, *n* = 63	0.54 ± 0.11	0.52 ± 0.10	0.3

Data are shown as mean ± SD; *p*-values by 2-tailed Student’s *t*-test. Abbreviations as in Table 1.

## Abbreviations

ACEI	angiotensin-converting enzyme inhibitors
ADMA	asymmetric dimethylarginine
ANCOVA	analysis of covariance
ANOVA	analysis of variance
CAD	coronary artery disease
CYP	cytochrome P450
DDAH-1	type 1 dimethylarginine dimethylaminohydrolase
ELISA	enzyme-linked immunosorbent assay
GFR	glomerular filtration rate
HDL	high-density lipoproteins
HO-1	type 1 heme oxygenase
LDL	low-density lipoproteins
NADPH	nicotinamide adenine dinucleotide phosphate
NO	nitric oxide
PPI	proton pump inhibitors
PRMTs-I	type I protein-arginine *N*-methyltransferases
SD	standard deviation
